# Directional Local Field Potentials in the Subthalamic Nucleus During Deep Brain Implantation of Parkinson’s Disease Patients

**DOI:** 10.3389/fnhum.2020.521282

**Published:** 2020-09-30

**Authors:** T. A. Khoa Nguyen, Michael Schüpbach, André Mercanzini, Alain Dransart, Claudio Pollo

**Affiliations:** ^1^Department of Neurosurgery, University Hospital of Bern, Bern, Switzerland; ^2^Department of Neurology, University Hospital of Bern, Bern, Switzerland; ^3^Microsystems Laboratory 4, School of Engineering, EPF Lausanne, Lausanne, Switzerland; ^4^Aleva Neurotherapeutics SA, Lausanne, Switzerland

**Keywords:** deep brain stimulation, local field potentials, segmented leads, subthalamic nucleus, Parkinson’s disease

## Abstract

Segmented deep brain stimulation leads feature directional electrodes that allow for a finer spatial control of electrical stimulation compared to traditional ring-shaped electrodes. These segmented leads have demonstrated enlarged therapeutic windows and have thus the potential to improve the treatment of Parkinson’s disease patients. Moreover, they provide a unique opportunity to record directional local field potentials. Here, we investigated whether directional local field potentials can help identify the best stimulation direction to assist device programming. Four Parkinson’s disease patients underwent routine implantation of the subthalamic nucleus. Firstly, local field potentials were recorded in three directions for two conditions: In one condition, the patient was at rest; in the other condition, the patient’s arm was moved. Secondly, current thresholds for therapeutic and side effects were identified intraoperatively for directional stimulation. Therapeutic windows were calculated from these two thresholds. Thirdly, the spectral power of the total beta band (13–35 Hz) and its sub-bands low, high, and peak beta were analyzed *post hoc*. Fourthly, the spectral power was used by different algorithms to predict the ranking of directions. The spectral power profiles were patient-specific, and spectral peaks were found both in the low beta band (13–20 Hz) and in the high beta band (20.5–35 Hz). The direction with the highest spectral power in the total beta band was most indicative of the 1^st^ best direction when defined by therapeutic window. Based on the total beta band, the resting condition and the moving condition were similarly predictive about the direction ranking and classified 83.3% of directions correctly. However, different algorithms were needed to predict the ranking defined by therapeutic window or therapeutic current threshold. Directional local field potentials may help predict the best stimulation direction. Further studies with larger sample sizes are needed to better distinguish the informative value of different conditions and the beta sub-bands.

## Introduction

Deep brain stimulation (DBS) of the subthalamic nucleus is an effective therapy for medically refractory cases of Parkinson’s disease (Deuschl, 2006; Weaver, 2009). Over 100,000 Parkinson’s disease patients have received a DBS implant (Strauss et al., 2014). Besides manageable surgical risks, DBS of the subthalamic nucleus carries the risk of stimulation-induced side effects. Even though modern stereotactic surgery can achieve sub-millimeter targeting accuracy (Nowacki et al., 2017), deviations of about 2 mm or more were reported for other surgical approaches (Guo et al., 2007; Lee et al., 2018). These deviations increase the likelihood of stimulation-induced side effects, which can affect up to 50% of implanted patients (Volkmann et al., 2009).

Directional stimulation with segmented leads is a promising approach to address this issue (Contarino et al., 2014; Pollo et al., 2014; Nguyen et al., 2019a). It can reduce undesired activation of adjacent structures such as the internal capsule, and indeed, it has demonstrated higher side effect thresholds than classical omnidirectional stimulation in implanted patients (Steigerwald et al., 2016; Dembek et al., 2017). Directional stimulation may further be leveraged to target very specific and clinically effective regions of the subthalamic nucleus, also known as “sweet spots” obtained from probabilistic mapping (Horn et al., 2017; Dembek et al., 2019; Nguyen et al., 2019b).

These regions are associated with abnormal electrophysiological activity recorded through local field potentials (LFPs). Specifically, spectral analysis has highlighted excessive oscillations in the beta band between 13 and 35 Hz. These were found to be correlated with the severity of motor symptoms (Hammond et al., 2007; Chen et al., 2010; Little et al., 2012) and were reduced by effective DBS (Kühn et al., 2008). With segmented leads, stimulation in the direction with the highest spectral power in the beta frequency band was associated with better motor improvement (Bour et al., 2015) or a wider therapeutic window (Tinkhauser et al., 2017a). Both these studies recorded LFP intraoperatively with patients at rest. However, motor tasks have been shown to decrease the power in the beta frequency band (Kühn et al., 2004) or modulate specific sub-bands (Tinkhauser et al., 2019) and may, therefore, provide additional guidance on identifying the best stimulation direction.

Here, we report directional LFPs from the subthalamic nucleus. Local field potentials were recorded intraoperatively with a segmented lead in two conditions: (i) with patients at rest and (ii) with passive movement of the patient’s arm. By analyzing the spectral power in the total beta frequency band and its sub-bands, we investigated whether and how the resting and moving conditions are indicators of the best stimulation direction. As a first step, this could guide the complex programming of directional DBS (ten Brinke et al., 2018).

## Materials and Methods

### Patient Recruitment, Surgical Procedure, and Intraoperative Assessment

Four patients with Parkinson’s disease were included in this study at the University Hospital of Bern. Two patients were female; ages ranged from 33 to 70 years with a median age of 62 years. The local ethics committee and the Swiss Competent Authority approved the study protocol, which conformed to the Good Clinical Practice guidelines and the International Organization for Standardization 14155 standard. All four patients provided written informed consent and represented a subset of our previous study (Pollo et al., 2014), who in addition agreed to LFP recordings. These were patients 9, 11, 12, and 13 in the previous study and were relabeled here as patients 1–4 (Table 1).

**TABLE 1:** Ranking of directions as defined by therapeutic window for the four patients.

Patient	1^st^ best direction (mA)	2^nd^ best direction (mA)	3^rd^ best direction (mA)
1	Antero-lateral (2.7, 0.5–3.2)	Medial (2.7, 0.5–3.2)	Postero-lateral (2.1, 0.4–2.5)
2	Antero-lateral (2.1, 0.4–2.5)	Postero-lateral (1.3, 0.6–1.9)	Medial (1.3, 0.9–2.2)
3	Medial (2.4, 1.0–3.4)	Antero-lateral (1.6, 1.7–3.3)	Postero-lateral (0.7, 1.6–2.3)
4*	Medial (2.7, 0.6–3.3)	Antero-lateral (2.4, 0.6–3.0)	Postero-lateral (1.7, 0.6–2.3)

The first value in brackets is the therapeutic window, followed by therapeutic current threshold and side effect threshold, all in mA. Rigidity was the main symptom for all patients. *Therapeutic current thresholds were identical for this patient. When ranking the directions by therapeutic current threshold, the therapeutic window was used as a tiebreaker (i.e., larger window ranks higher).

The patients were implanted under local anesthesia with a stereotactic frame (Leksell frame, Elekta, Stockholm, Sweden). For surgical planning, preoperative magnetic resonance images were coregistered to stereotactic computer tomography images. They received implantation in both hemispheres, but testing with the segmented lead was performed only in the first hemisphere operated on.

Firstly, microelectrode recording and macrostimulation were performed to refine the location of the subthalamic nucleus and to identify the target trajectory and depth for permanent implantation.

**FIGURE 1:**
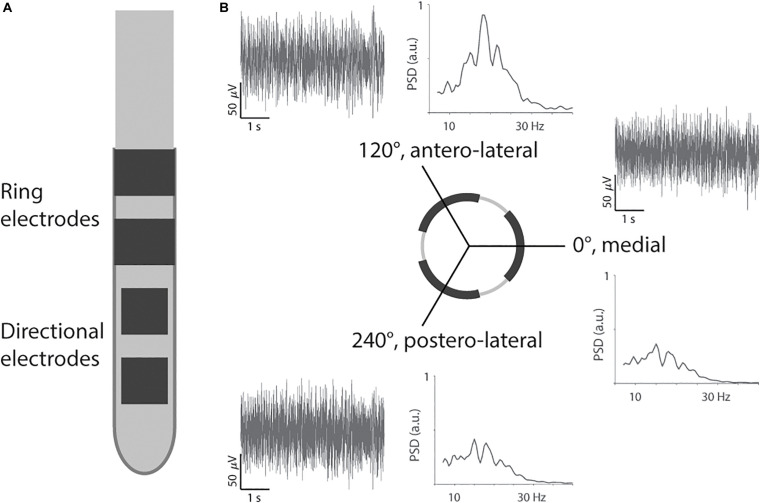
Segmented lead and recording of directional local field potentials. **(A)** The segmented lead had two levels of directional electrodes and two levels of ring electrodes. Only the most distal level of directional electrodes toward the tip was used for recording and stimulation. **(B)** Example of filtered traces and power spectral densities (PSD) from Patient 2 for the three directions.

Secondly, the segmented lead was inserted (directSTN Acute, Aleva Neurotherapeutics, Lausanne, Switzerland, Figure 1A). This lead was specifically designed for the intraoperative study and featured two levels of directional electrodes (electrode surface area of 1 mm^2^). The lead was placed in the same trajectory as intended for the permanent lead, and the distal level of directional electrodes was lowered to the same depth as in the previous step of macrostimulation. The segmented lead’s first directional electrode at 0° was oriented toward medial, the second electrode at 120° toward antero-lateral, and the third electrode at 240° toward postero-lateral. The surgeon used a marker line at 0° along the lead for orientation and inserted the lead without extra rotation. The depth of the segmented lead was intraoperatively confirmed with fluoroscopy.

Thirdly, the segmented lead was used for intraoperative LFP recording. The three directional electrodes on the distal level were used to record LFP simultaneously for 30 s. The electrical return was set to the cannula, 19 mm away from the recording contacts (pseudomonopolar montage). The LFPs were recorded for a resting condition (i.e., patient at rest) and then a passive moving condition (i.e., extension and flexion of the elbow) after a short break of 5 s. However, this study did not include instrumentation to synchronize movement and LFP recording. The LFPs were acquired with the Medtronic Leadpoint system (Medtronic, Fridley, MN, United States) with a sampling rate of 24 kHz for each direction. The Leadpoint system was additionally configured to apply a band-pass filter between 5 Hz and 5 kHz, and a notch filter at 50 Hz. Inserting the segmented lead after microelectrode recording should reduce the impact of a stun effect on the LFP recording (Chen et al., 2006). In addition, we performed LFP recordings at one depth only, and therefore, the impact of a stun effect should be similar for the three directions.

Fourthly, the segmented lead was used for intraoperative clinical testing. It was connected to an external neurostimulator with multiple independent current-driven sources (Osiris Stimulators, Model 504196, Inomed GmbH, Emmendingen, Germany). Each of the three directional electrodes was tested with monopolar stimulation. The stimulation pulses were cathodic-first with a pulse width of 90 μs and frequency of 130 Hz. A metal plate was used as a distant current return and placed in the subclavicular area, similar to the area for the permanent implantable pulse generator. A separate operator configured the stimulation direction and current amplitude, and thus, the patient and the clinician assessing the clinical effects of stimulation were blinded to the stimulation configuration.

The clinical effects were assessed based on the rigidity of the patient’s hand. The assessment was performed by the same experienced neurologist for all four patients. The stimulation current was increased in 0.2 mA steps to determine the therapeutic current threshold, i.e., the lowest stimulation current that resulted in no rigidity. Then, the stimulation current was further increased to determine the side effect current threshold, where a sustained side effect such as paresthesia, dysarthria, or focal muscular contraction was observed. The difference between the two thresholds was defined as the *therapeutic window* and was used to rank the directions. The direction with the largest window was ranked as 1^st^ best direction and so on. When directions had the same therapeutic window, the direction with the lower therapeutic current threshold was ranked higher. Therapeutic current thresholds, side effect current thresholds, and therapeutic windows for all patients and directions are listed in Table 1.

Finally, the segmented lead was removed after clinical testing, and the permanent lead was inserted using the same guide tube.

### Data Analysis

In our analysis, we first analyzed the spectral power of LFP recordings for the two conditions. Then, the spectral power was used by different algorithms to predict the ranking of directions.

The LFP recordings were analyzed *post hoc* with Matlab 2019b (MathWorks, Natick, MA, United States). Firstly, the 30-s recordings were downsampled to 375 Hz and visually inspected for artifacts. The resting recording for patient 3 was shortened to the period from 0 to 25 s due to an artifact at the end. All other recordings were processed at full length. Power spectral densities were then computed with the Welch method with 25% overlap and a spectral frequency resolution of 0.5 Hz. The spectral power of the beta band was calculated as the area under the density curve between 13 and 35 Hz (Figure 1B). Furthermore, we calculated the power in three sub-bands: low beta (13–20 Hz), high beta (20.5–35 Hz), and peak beta, i.e., 2 Hz around the beta peak (Tinkhauser et al., 2017a, the peak was determined by the maximum power spectral density across the three directions). The spectral power was normalized to the average power in the beta frequency band across the three recorded directions (Geng et al., 2018). This normalization was done separately for the resting and moving conditions. For the comparison of movement condition versus resting condition, the normalization factor from the moving condition was applied to both conditions.

We benchmarked different algorithms to predict the ranking of directions. The *actual* ranking was determined from the intraoperative testing as described above, i.e., the direction with the largest therapeutic window was ranked as 1^st^ best direction and so on. In addition, we also tested the therapeutic current thresholds for the ranking. The direction with the lowest threshold was ranked as 1^st^ best direction and so on. The *predicted* ranking was determined from the spectral power, i.e., the direction with the highest spectral power was predicted as 1^st^ best direction and so on. For therapeutic current thresholds, initial prediction performances were low, and we additionally tested the reverse order, i.e., the lowest spectral power was predicted as 1^st^ best direction and so on. The different algorithms were based on the spectral power for the moving condition and resting condition, as well as the ratio of moving-to-resting and for the four sub-bands, total, low, high, and peak beta. Confusion matrices were used to illustrate the prediction performance of an algorithm.

## Results

### Power Spectral Densities for Different Conditions and Directions

We recorded directional LFPs through a segmented lead in four Parkinson’s disease patients undergoing subthalamic nucleus implantation.

**FIGURE 2:**
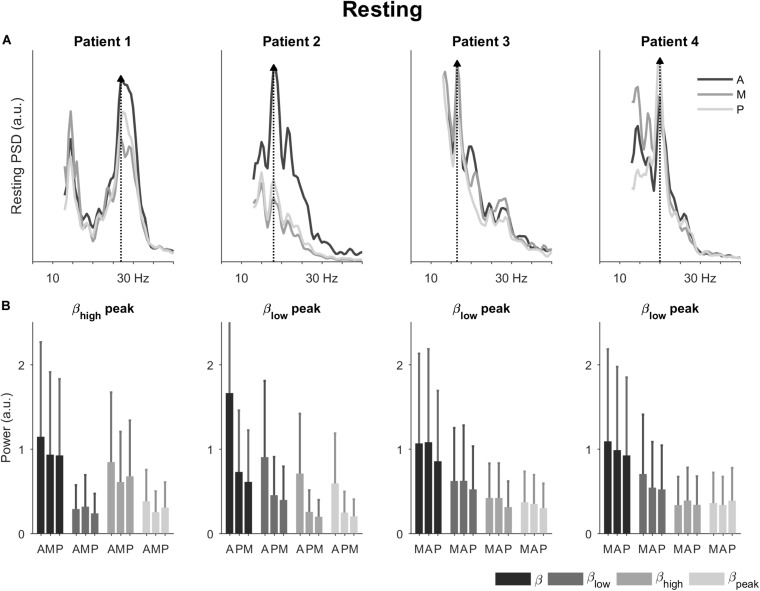
Power spectral densities and power in the resting condition. **(A)** Power spectral densities for the four patients highlighting the direction with peak spectral density (dotted line and filled triangle). **(B)** Power for the four patients in the total beta band and its sub-bands. The bars are ordered by 1^st^, 2^nd^, and 3^rd^ best direction for each patient (Table 1). The error bars represent the 95% confidence interval. A, antero-lateral; M, medial; P, postero-lateral directions.

In the resting condition, the power spectral densities showed different profiles for each patient (Figure 2A). Patient 1 had a spectral peak in the high beta band; and patients 2, 3, and 4 had a spectral peak in the low beta band (27, 18, 16.5, and 20 Hz, respectively). The direction with the peak spectral density was antero-lateral for patients 1 and 2 and medial for patients 3 and 4.

Also in the resting condition, we observed differences in power for the three directions (Figure 2B). The most noticeable differences were observed for patient 2. For this patient, the antero-lateral direction had the highest power in the total beta band followed by the postero-lateral and medial directions. This directional ranking was consistent across the sub-bands. For the other patients, differences in power were less marked between the directions. Generally, the direction with the peak spectral density had also the most power across the total beta band. None of the sub-bands emphasized directional differences in particular.

**FIGURE 3:**
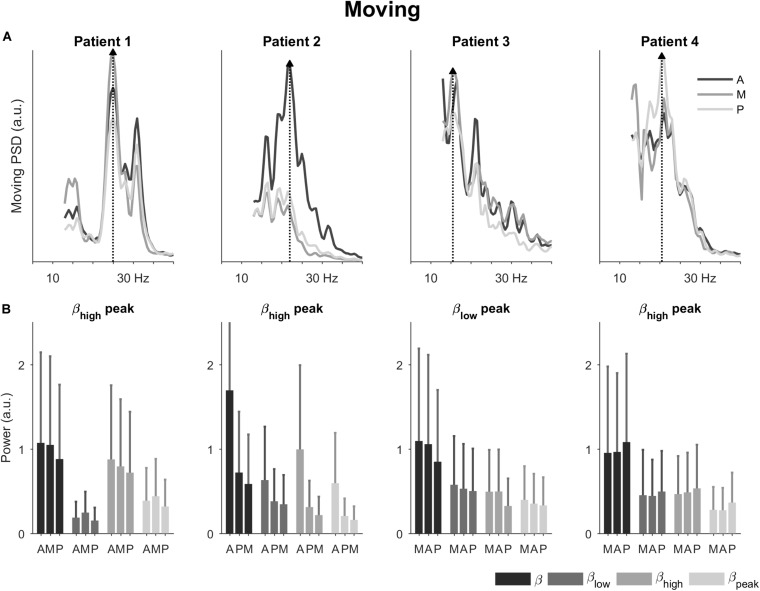
Power spectral densities and power in the passive moving condition. **(A)** Power spectral densities for the four patients highlighting the direction with peak spectral density (dotted line and filled triangle). The peaks shifted slightly in patients 1, 2, and 4 compared to the resting condition. **(B)** Power for the four patients in the total beta band and its sub-bands. The bars are ordered by 1^st^, 2^nd^, and 3^rd^ best direction for each patient (Table 1). The error bars represent the 95% confidence interval. A, antero-lateral; M, medial; P, postero-lateral directions.

In the passive moving condition, the power spectral densities remained patient-specific and were accompanied by a slight shift in spectral peaks (Figure 3A). Now, patients 1, 2, and 4 had spectral peaks in the high beta band, whereas patient 3’s spectral peak remained in the low beta band (25, 22, 20.5, and 15.5 Hz, respectively). The directions with the peak spectral density were the same as in the resting condition, except for patient 1 where a change from antero-lateral to medial direction was observed.

In terms of power (Figure 3B), directional trends in the moving condition were similar to the ones in the resting condition. The most noticeable directional differences were again observed for patient 2. In patient 1, the power for the medial direction became more prominent compared to the resting condition.

**FIGURE 4:**
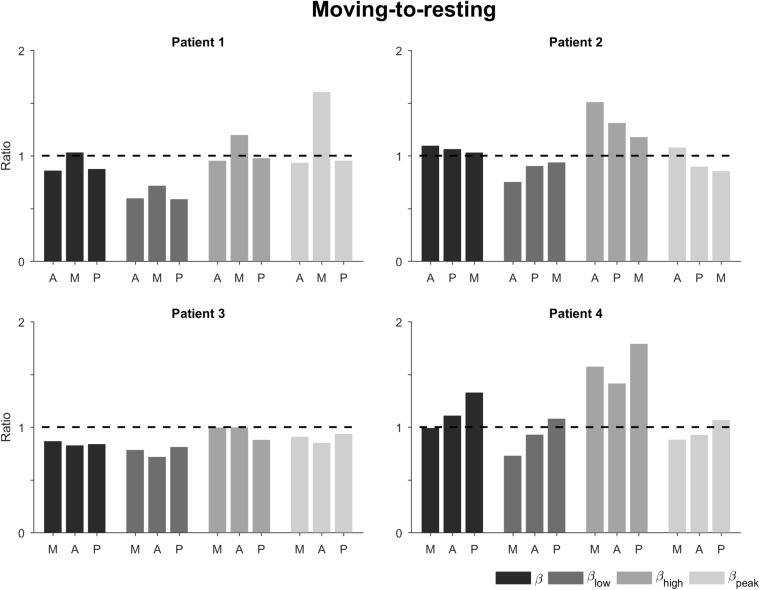
Ratio of moving-to-resting power for the different patients, directions, and sub-bands. The bars are ordered by 1^st^, 2^nd^, and 3^rd^ best direction for each patient (Table 1). The dashed horizontal line indicates no change between moving and resting. A, antero-lateral; M, medial; P, postero-lateral directions.

Calculating the power ratio of moving-to-resting condition showed some changes in the beta band and its sub-bands (Figure 4). The ratios for the total beta band indicated only minor changes between the conditions (i.e., ratio of 1). The ratios for the low beta band indicated a decrease in power in the movement condition (i.e., ratio <1). This decrease was most marked for patients 1 and 3. The ratios for the high beta band, on the other hand, indicated an increase in power in the movement condition (i.e., ratio >1). This was most noticeable in patients 2 and 4. The power in the peak beta band indicated an increase in power in the movement condition for Patient 1 and only small changes for the other patients. In terms of directions, the ratios did not show a tendency.

**FIGURE 5:**
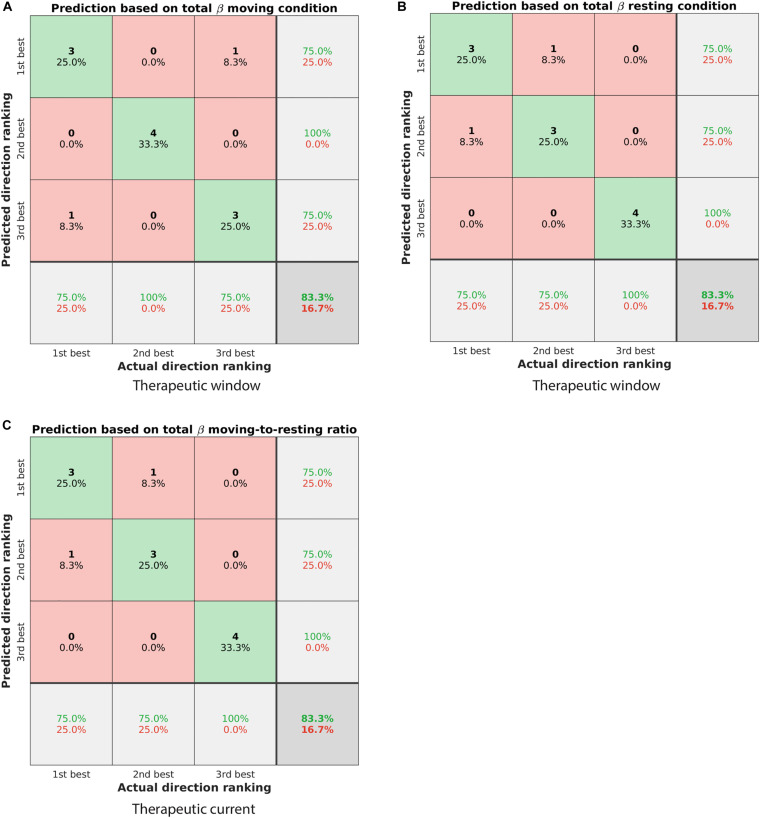
Confusion matrices showing the classification performance of different ranking algorithms. **(A)** Predicting the ranking of directions based on the spectral power in the total beta band of the moving condition. The predicted ranking was done from highest to lowest spectral power. The actual ranking was defined by the therapeutic window. Elements on the diagonal from top left to bottom right illustrate correctly classified directions (green shaded), whereas other elements were incorrectly classified (red shaded). In total, 83.3% of directions were classified correctly. **(B)** Similar to panel **(A)** but for the resting condition. **(C)** Predicting the ranking of directions as defined by the therapeutic current threshold. The prediction was based on the spectral power ratio moving-to-resting in the total beta band, but from lowest to highest spectral power ratio (cf. Table 2).

### Spectral Power to Predict Direction Ranking

For the therapeutic window, the spectral power in the total beta band was the best predictor to rank the three directions. Specifically, ranking the directions from highest to lowest spectral power in that band generally matched the ranking of 1^st^, 2^nd^, and 3^rd^ best direction. Both the moving condition and the resting condition were similarly predictive and classified 83.3% of the directions correctly (Figures 5A,B). For instance, the direction with the highest spectral power in the total beta band matched the 1^st^ best direction in three out of four patients.

For the therapeutic current threshold, a different ranking algorithm was the best predictor. In contrast to the ranking above, we found ranking the directions from lowest to highest spectral power to be more predictive of the therapeutic current threshold. Specifically, the spectral power *ratio* of moving-to-resting from the total beta band was most predictive and classified 83.3% of the directions correctly (Figure 5C). For instance, the direction with the lowest spectral power ratio in that band matched the 1^st^ best direction in three out of four patients. The percentages of correctly and incorrectly classified directions are summarized for all conditions and sub-bands in Table 2.

**TABLE 2:** Classification performance of the different algorithms.

Algorithm	Correctly classified (%)	Incorrectly classified (%)	Algorithm	Correctly classified (%)	Incorrectly classified (%)
**Ranking high-to-low spectral power**			**Ranking high-to-low spectral power**		
**Window, moving**			**Current, moving**		
**Total beta**	**83.3**	**16.7**	Total beta	16.7	83.3
Low beta	58.3	41.7	Low beta	16.7	83.3
High beta	66.7	33.3	High beta	25	75
Peak beta	58.3	41.7	Peak beta	16.7	83.3
**Window, resting**			**Current, resting**		
**Total beta**	**83.3**	**16.7**	Total beta	41.7	58.3
Low beta	66.7	33.3	Low beta	50	50
High beta	41.7	58.3	High beta	25	75
Peak beta	58.3	41.7	Peak beta	16.7	83.3
**Window, moving-to-resting**			**Current, moving-to-resting**		
Total beta	41.7	58.3	Total beta	25	75
Low beta	25	75	Low beta	41.7	58.3
High beta	33.3	66.7	High beta	16.7	83.3
Peak beta	33.3	66.7	Peak beta	16.7	83.3
			**Ranking low-to-high spectral power**		
			**Current, moving**		
			Total beta	66.7	33.3
			Low beta	66.7	33.3
			High beta	58.3	41.7
			Peak beta	66.7	33.3
			**Current, resting**		
			Total beta	41.7	58.3
			Low beta	58.3	41.7
			High beta	33.3	66.7
			Peak beta	41.7	58.3
			**Current, moving-to-resting**		
			**Total beta**	**83.3**	**16.7**
			Low beta	66.7	33.3
			High beta	41.7	58.3
			Peak beta	66.7	33.3

*Best performing classifiers are set in bold and are also shown in Figure 5. Left part of the table using the therapeutic window for the actual ranking (larger window—higher ranking); right part of the table using the therapeutic current threshold for the actual ranking (lower threshold—higher ranking).*

## Discussion

This study investigated directional LFPs from the subthalamic nucleus and their potential to determine clinically effective stimulation directions. The study emphasized three findings. Firstly and most importantly, we found that ranking directions from highest to lowest spectral power in the *total beta band* generally mirrored the ranking of best stimulation directions as defined by therapeutic window. For instance, the direction with the highest spectral power was also the 1^st^ best stimulation direction in three out of four patients. Our finding is in agreement with previous studies that recorded directional LFPs with patients at rest. These reported better motor improvement or wider therapeutic window for stimulation in the direction of highest beta activity (Bour et al., 2015; Tinkhauser et al., 2017a). The direction with the highest beta activity may point toward a highly pathological cell cluster in the subthalamic nucleus that limits information coding in the motor network of the brain (Little and Brown, 2014). Stimulation in that direction may suppress beta activity as has been shown with omnidirectional leads (Kühn et al., 2008) and segmented leads (Bour et al., 2015). This in turn may release information flow in the motor network as demonstrated in computational simulations (Humphries et al., 2018; Müller and Robinson, 2018) and thus improve motor symptoms.

Interestingly, to predict the ranking of directions as defined by therapeutic current threshold required a different ranking algorithm. This algorithm ranked directions from lowest to highest spectral power in the total beta band and used the power ratio of moving-to-resting. A lower spectral power in one direction may indicate a relatively smaller pathological cell cluster compared to the other directions, which in turn may require a lower current amplitude to release information flow in the motor network. Furthermore, a lower spectral power ratio of moving-to-resting suggests that this direction with the smaller pathological cell cluster is more sensitive to movement modulation than the other directions.

Secondly, our main finding about ranking based on the total beta band was valid both for the resting condition and for the moving condition. With respect to spectral power, there was no clear difference in the total beta band between the resting and moving conditions (Figures 4, 5). Two patients showed a decrease in the low beta band in response to movement, whereas the two other patients showed an increase in the high beta band. With *active* movement, beta activity has been found to decrease prior to movement and increase again after movement (Levy et al., 2002; Kühn et al., 2004). With *passive* movement like in this study, beta activity has been found to be movement-locked with a decrease at the time of maximum angle of the elbow (Connolly et al., 2015). Our results are in accordance with these findings, even if the protocol of this exploratory study did not include synchronization of LFP recording and movement of the joint. Thus, we were not able to precisely analyze temporal characteristics, which may have added directional information as has been recently suggested in non-human primates (Zhang et al., 2018) or patients (Tinkhauser et al., 2019).

Thirdly, we analyzed three beta sub-bands and observed beta band profiles that were patient-specific with spectral peaks in the low or high beta band. The beta band was divided into a low and a high beta band previously to discuss different influences (Priori et al., 2004). Spectral peaks in the low beta band were significantly reduced by acute antiparkinsonian medication (levodopa), while activity in the high beta band was affected by movement (Kühn et al., 2004; Tinkhauser et al., 2019). The divergence of peaks in our cohort may help explain why none of the sub-bands provided additional directional information. The divergence also seems to agree with a previous study suggesting that the beta band profile was a patient-specific “fingerprint” (Bronte-Stewart et al., 2009). This view has been recently supported by a computational study of LFPs (Maling et al., 2018).

Several limitations of our study are noteworthy. Our recordings were performed at one depth only due to intraoperative constraints, but the beta band profile is known to change along the subthalamic nucleus (Bour et al., 2015; Horn et al., 2017; Geng et al., 2018). We only tested patients with rigidity-dominant Parkinson’s disease, although LFPs were reported to be symptom-specific (Fernández-García et al., 2017; Telkes et al., 2018). Our exploratory study examined the averaged power in the beta band across 30-s recordings. It did not analyze beta bursts that may reveal further information about the best stimulation direction and may be additionally relevant for adaptive DBS to switch directions or modulate the stimulation amplitude (Little et al., 2016; Tinkhauser et al., 2017b, 2018). Future studies therefore need to shed more light on the beta band or beta burst profile of Parkinson’s disease. In the meantime, the total beta band may be the most robust indicator of best stimulation direction.

In summary, directional LFPs recorded with segmented leads support the hypothesis that spectral power may be indicative of the best stimulation direction. More specifically, the spectral power in the total beta band was the best predictor of direction ranking in our small cohort. This preliminary finding needs to be confirmed in a future study with a larger patient cohort. A refined analysis of the movement and resting conditions should provide a better understanding of the patient-specific beta band profile.

## Data Availability Statement

The datasets generated for this study are available on request to the corresponding author.

## Ethics Statement

The studies involving human participants were reviewed and approved by the Ethical Commission of the Canton Bern (KEK), number (KEK 072-12 or ClinicalTrials.gov identifier NCT01764815). The patients/participants provided their written informed consent to participate in this study.

## Author Contributions

TN contributed to the data analysis, visualization, drafting of the manuscript, and editing. MS contributed to the data curation and manuscript review. AM contributed to the conceptualization and data curation. AD contributed to the data curation. CP contributed to the conceptualization, data curation, supervision, manuscript review, and editing. All authors contributed to the article and approved the submitted version.

## Conflict of Interest

TN was previously an employee of Aleva Neurotherapeutics SA and has no financial stake with the company. Currently, he is supported by a grant from the Swiss National Science Foundation (PZ00P3_186142). MS has received financial support from Boston Scientific, but not related to this study. AM is a co-founder of Aleva Neurotherapeutics. AD is an employee of Aleva Neurotherapeutics. CP is a co-founder of Aleva Neurotherapeutics and did not receive any honorarium or consulting fees for this study from Aleva Neurotherapeutics. He has received consulting fees from Boston Scientific, but not related to this study.
